# Lichen Planopilaris: The first biopsy layer microbiota inspection

**DOI:** 10.1371/journal.pone.0269933

**Published:** 2022-07-18

**Authors:** Daniela Pinto, Francesco Maria Calabrese, Maria De Angelis, Giuseppe Celano, Giammaria Giuliani, Fabio Rinaldi

**Affiliations:** 1 Human Advanced Microbiome Project-HMAP, Milan, Italy; 2 Department of Soil, Plant and Food Science, “Aldo Moro” University, Bari, Bari, Italy; Universidad Nacional Autonoma de Mexico Facultad de Quimica, MEXICO

## Abstract

Lichen Planopilaris (LPP) is a lymphatic disease affecting the scalp that is characterized by a chronic and destructive inflammation process, named as ‘cicatricial alopecia’ in which the hair follicles are targeted and may involve predominantly lymphocytes or neutrophils. Scalp and biopsy layers have never been used to investigate microbial community composition and its relative taxa abundances in LPP. We sought to examine the significant taxa of this chronic relapsing inflammatory skin disease, together with inspect the existing connections with metabolic pathways featuring this microbial community. We used a multilevel analysis based on 16S rRNA marker sequencing in order to detect OTU abundances in pathologic/healthy samples, real time PCR for measuring the levels of IL-23 interleukin expression and urinary metabolomics to find out volatile organic metabolites (VOMs). By using a linear regression model, we described peculiar taxa that significantly differentiated LPP and healthy samples. We inspected taxa abundances and interleukin mRNA levels and the *Microbacteriaceae* family resulted negatively correlated with the IL-23 expression. Moreover, starting from 16S taxa abundances, we predicted the metabolic pathways featuring this microbial community. By inspecting microbial composition, sample richness, metabolomics profiles and the relative metabolic pathways in a cohort of LPP and healthy samples we deepened the contribution of significant taxa that are connected to inflammation maintenance and microbiota plasticity in LPP pathology.

## 1. Introduction

The Scalp possesses unique features (high follicular density and sebum production) that make it susceptible to several conditions including the possibility to undergo to an inflammatory status [1]. Psoriasis and seborrheic dermatitis are the most frequent pathologies [2] and their manifestations could be often similar in the initial steps that specifically include erythema, skin scaling or desquamation, and pruritus.

Other less frequent inflammatory conditions of the scalp can mimic the most common forms [3]. Among them, Lichen Planopilaris (LPP) occurs when Lichen Planus, a common skin disease involves the scalp, especially the vertex area [4]. First described in 1895 and also named as “follicular lichen planus of the scalp” it usually manifests in the form of irregular cicatricial alopecia, always irreversible [5].

According to the North American Hair Research Society (NAHRS) LPP is now classified as a primary lymphocytic disease based on lymphocytic, neutrophilic, or mixed infiltrates [6].

Noticeably, few data are nowadays available as regards of its epidemiology. Authors [7] reported higher frequency in Caucasians and Indians whereas lower incidence was found for Asians. Looking at gender ratio, females result more affected than males (1.8:1) with a typical presentation occurring between 40–60 years [8].

Based on differentiated symptoms, three different variants of LPP can be observed. The first one usually involves the vertex and is characterized by a perifollicular violaceous erythema and keratotic plugs that in some cases can be accompanied by inflammation and hyperkeratosis [4]. The second variant is known as Frontal Fibrosing Alopecia (FFA); it is characterized by a distinctive pattern of a progressive recession on frontal hairline and eyebrow, scalp pruritus, and perifollicular erythema [9]. This signs mostly affect middle-aged women. The so-called Graham-Little Piccardi Lassueur Syndrome is reported as the third LPP variant; its common manifestations are patchy cicatricial alopecia on the scalp, non-cicatricial alopecia on the axilla and groin, and the formation of the so called ‘follicular spinous papule’ on the body and/or scalp [10].

More recently, a fourth variant has been included in the list of sub variants: Fibrosing Alopecia in a Pattern Distribution (FAPD). It occurs in patients affected by Androgenetic Alopecia (AGA), that present also the typical lesions of LPP [11].

Regardless of the type of variant, LPP evolves in a very extensive and active way, up to involving the entire scalp and leading to irreversible baldness [12, 13]. In this clinical picture, common clinical manifestations include perifollicular erythema and hyperkeratosis, persisting itching on the scalp, scaling, and enduring painful sensation on the scalp, namely ‘trichodynia’ [14]. Compared to AGA, the hair count per follicular unit is found to be lower, probably as result of the follicular dropout which happens as a consequence of a cicatricial event [15].

Nowadays LPP diagnosis is based on clinical and histopathological findings and on an accurate differential diagnosis that mainly evidences the presence of seborrheic dermatitis, psoriasis and AGA at an initial stage [16]. Due also to poor information on physiopathology and a not fully understood etiology, a complete and accurate diagnosis of LPP remains an open challenge.

Its clinical expression has been reported to be triggered mainly by cell-mediated immunity [17]. The immune response mostly involves the bulge area, a contiguous part of outer root sheath enriched in stem cells, with the engagement of T lymphocytes (CD4 and CD) that are activated by the increasing of Langerhans cells both in the dermis and epidermis. The Th17 (subset of CD4+ T helper) cells has been recently reported to play a crucial role in promoting immune-inflammatory reactions also in autoimmune diseases [18] and some of their secreted cytokines are involved in the defense against pathogenic microorganisms and can correlate with specific bacterial genera in Oral Lichen Planus [19].

A link between LPP and the microbial population inhabiting the scalp has been also recently reported [20]. As reported for the gut [21] and skin microbiome [22], bacteria and fungi are strongly involved in the healthy status, and the host immune system is recognized as a major stress able to modulate the microbial composition [23].

Perturbation in host immune response as well as the alteration of the scalp microbiota observed in our previous study on another model of scalp auto-immune disease [24], may suggest a disequilibrium within the scalp microbiota in LPP patients and a role in its physiopathology.

To test this hypothesis, we studied the scalp bacterial abundance and diversity, as well as distinct scalp metagenomic profiles, cytokine profile, and urine metabolite expression in subjects affected by LPP compared to healthy subjects. We also explored the relationships between clinical manifestations and bacterial microbiota also in terms of predicted microbial biochemical pathways and we inspected the urine metabolomics profiles of LPP patients. Analyses were conducted both on the scalp and the subepidermal scalp compartments.

## 2. Material and methods

### Study design and participants

A cohort of 27 LPP and healthy subjects (people never showing the symptoms related to any dermatologic disease) of both sex (six samples for each one of the three biopsy subepidermal compartments plus eleven lichen swabs), aged between 18–60 years, were enrolled. LPP patients were diagnosed clinically and confirmed as having LPP by biopsy, according to the WHO criteria. Essential clinical data were collected at baseline under dermatological control.

Patients were evaluated for erythema and desquamation (scaling) using a 5-point scale: 0 = none, 1 = minimal, 2 = mild, 3 = moderate, and 4 = severe. Besides, the investigator assessed itching/pruritus over the past 24 hours using a visual analogue scale (VAS) from 0 = no pruritus to 10 = severe pruritus.

Patients from both groups were evaluated and enrolled in the study, after signed informed consent, by the RS Dermatologic Clinic, Milan, Italy.

The study was under the approval of the Ethical Independent Committee for Clinical, not pharmacological investigation in Genoa (Italy) and following the ethical standards of the 1964 Declaration of Helsinki. All the volunteers signed an informed consent.

Subjects had also to accept to not receive drug/cosmetic treatments during the study. Exclusion criteria include pregnancy or breastfeeding; any other medical condition or other scalp or hair disorders; last shampoo performed 48 h before sampling; no anti-tumor, immunosuppressant, or radiation therapy in the last 3 months; no antibiotic in the last 30 days before sampling; no presence of underlying infection; no probiotic in the last 15 days; no topical or hormonal therapy on the scalp in the last 3 months.

### Subject recruitment and sample collection

Enrolled subjects were sampled through swab procedure according to previously reported methods [24]. eNAT^™^ kit (1 ml eNAT^™^ transport and preservation medium and FLOQSwab^™^) (Copan, Brescia, Italy) was used for sampling a 16 cm^2^ area. Samples were stored at 4°C until DNA extraction.

A 4-mm biopsy punch was also used to assess the microbial community in the subepidermal compartments of the scalp [24]. Some enrolled healthy subjects were already analyzed in a previously published cohort [24], and here reanalyzed for the metagenomic and metabolite profiles in urinary samples.

### DNA extraction and 16S amplicon generation, sequencing, and analysis-Illumina sequencing

Bacterial DNA from collected swabs was extracted by means of QIAamp Dneasy Tissue kit (Qiagen, Milan, Italy) according to manufacturer protocol, with minor modifications [25] following by quantification with QIAexpert system (Qiagen, Milan, Italy) before sequencing.

Following universal prokaryotic primers were used for the V3-V4 variable region: 341 F CTGNCAGCMGCCGCGGTAA [26, 27] and 806bR GGACTACNVGGGTWTCTAAT [28–30] at Personal Genomics (Verona, Italy) following the method of Caporaso et al. [31] and Kozich et al. [32], with minor modifications. The 300PE instrument (Illumina, San Diego, CA) was used for libraries generation. Bioinformatics analyses, from processing of raw fastQ files to alpha index estimates were conducted in QIIME2 [33] () microbiome platform (version 2020.8). QIIME plugin q2-deblur [34] was used for the 16S denoising step. The ad hoc customized classifier “gg-13-8-99-515-806-nb-classifier.qza”, was used to infer taxonomy. QIIME2 plugins were used to compute alpha diversity metrics including Shannon entropy and Faith’s PD.

### Predictive functional profiling of microbial LPP communities

16S bacteria rRNA gene sequences were the starting point for the prediction of metabolic functions in the LPP scalp microbiota by using PICRUSt—Phylogenetic Investigation of Communities by Reconstruction of Unobserved States [35]. Specifically, the make.biom command of the Mothur program, based on a Greengenes database [36], was used to obtain a BIOM-formatted OTU table. In order to reflect the true taxa abundance, each OTU was corrected by normalizing the 16S rRNA copy number. By running the Picrust “predict_metagenomes.py” script, KEGG orthology abundances for a given OTU were table-picked using the latest version of Greengenes database.

The gene functions classified by KO were further categorized into KEGG pathways using the “categorize_by_function.py” PICRUSt script, that collapses thousands of predicted functions into higher categories (KEGG pathways). A two-sided corrected (BH) Welch’s t-test (p < 0.05), within the STAMP software [37] was used to assess the enrichment of predicted KEGG pathways.

### Cytokine assay

DNA extracted from biopsy samples was used for quantitative real-time PCR (RT-PCR) on interleukin 23 (*IL-23*) gene. DNA was amplified with a Stratagene Mx3000P Real-Time PCR System (Agilent Technologies Italia S.p.A., Milan, Italy). Following Taqman gene expression assays were used: Hs00372324_m1 (*IL-23A*) and Hs999999 m1 (*GAPDH*, human glyceraldehyde-3-phosphate dehydrogenase). Human *GAPDH* was used as the housekeeping gene. PCR amplifications were carried out in a 20 μl total volume: 10 μl of 2 × Premix Ex Taq (Takara, Japan), 1 μl of 20 × TaqMan gene expression assay, 0.4 μl of RoX Reference Dye II (Takara, Japan), 4.6 μl of water, and 4 μl of DNA at following PCR conditions: 95°C for 30 s, followed by 40 cycles of 95°C for 5 s and 60°C for 20 s. PCR reactions were performed in duplicate. The relative abundance of the expression of each gene was calculated by comparing delta cycle thresholds.

### Volatile urinary metabolomics

Volatile organic metabolites (VOMs) from urine samples were evaluate as potential biomarkers in healthy and LPP subjects. Two grams of collected urine were supplied in a 20 ml glass vial and spiked with 10 μl of internal standard solution (2-pentanol-4-methyl) at 33 ppm. To obtain the best extraction efficiency, the solid phase microextraction (SPME) was performed by exposing a conditioned 75 μm Carboxen/PDMS fiber (Supelco, Bellefonte, PA, USA) to the headspace of 2 g of acidified (pH 2) urine sample with 1 g of NaCl for 60 min at 60°C after a 35 min incubation [38]. The e following step of extraction was carried out with a CombiPAL system injector autosampler (CTC Analytics). The extracted compounds were desorbed in splitless for 3 min at 280°C [39]. A Clarus 680 (PerkinElmer, Waltham, MA, USA) gas chromatograph equipped with an Elite-624Sil MS Capillary Column (30 m × 0.25 mm i.d., 1.4-μm film thickness; PerkinElmer) was used. The gas chromatography system was coupled to a single quadrupole mass spectrometer Clarus SQ 8C (Perkin Elmer). The source and transfer line temperatures were kept at 250 and 230°C, respectively. Electron ionization masses were recorded at 70 eV, and the mass-to-charge ratio interval was m/z 34 to 350. The obtained chromatogram was analyzed for peak identification using the National Institute of Standard and Technology 2008 (NIST) library. A peak area threshold >1,000,000 and 90% or a greater probability of matches was used for VOMs identification, followed by inspection of the fragment patterns when required. Quantitative data for the compounds identified were obtained by the interpolation of the relative areas vs. the internal standard area.

### Statistical analysis

Statistically significant differences in alpha diversity and bacterial communities were obtained by Welch’s t-test corrected by multiple tests (Benjamini-Hochberg). Differences between groups were reported only when significative. Significant taxa at different taxonomic levels were computed by using a regression linear model implemented in MaAsLin2 software (https://huttenhower.sph.harvard.edu/maaslin/).

## 3. Results

### Clinical evaluation and cytokine assay

Twenty-seven healthy or suffering from LPP subjects were enrolled in the study and the groups of lichen and healthy subjects have comparable demographic characteristics (S1 File, sheet 1).

Clinical manifestations typical of LPP were observed in erythema severity score (mean of 2.76±0.7), scaling (mean of 3.25±0.9) and itching/pruritus (mean of 7.15±1.9).

The expression of *IL-23* was measured by means of qRT-PCR and resulted higher in the LPP samples than healthy subjects (S1 Fig).

### Richness of the bacterial population in LPP

The complexity among the analyzed biopsy layers was estimated by the analysis of community richness. Regardless of the considered sub-epidermal layer, a higher alpha diversity in LPP versus healthy samples was detected both with Shannon entropy and Faith’s PD metrics (S2 Fig).

### Taxonomic differences among healthy individuals and LPP

Raw read fastQ files have been demultiplexed and denoised in Qiime2 (S1 File, sheets 2–3).

**Fig 1:**
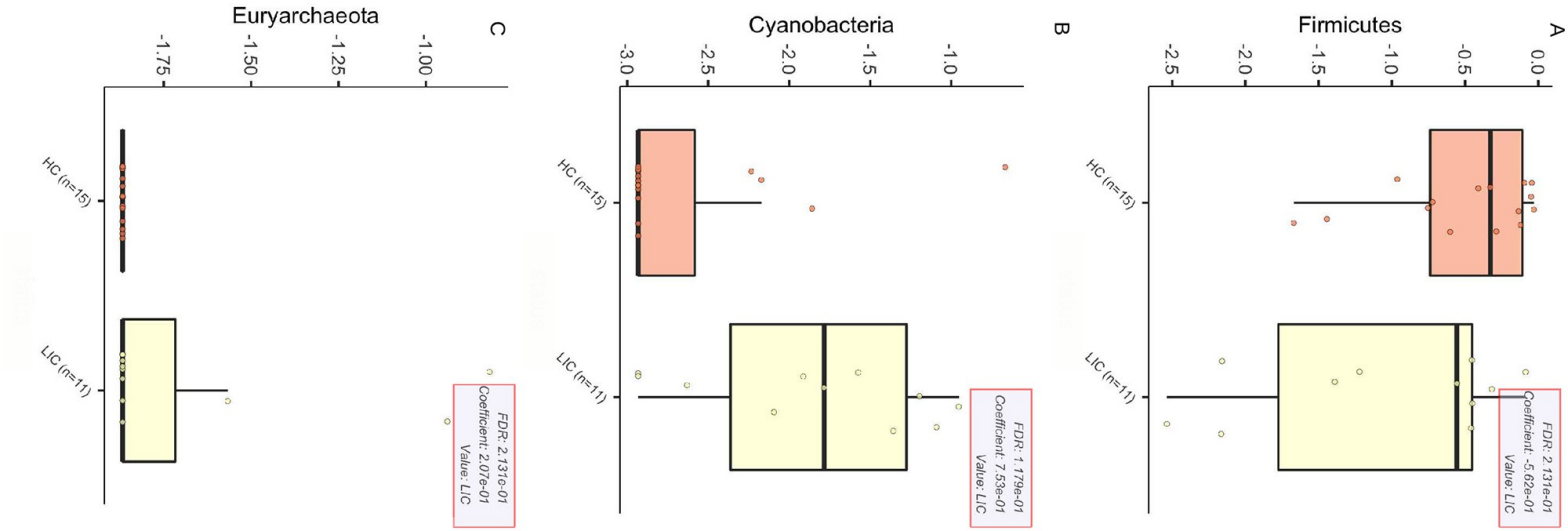
Statistically significant phyla that differed in the LPP swab. The three panels report all the statistically different phyla found by applying the regression linear model implemented in MaAsLin2 software. Metadata group information were defined considering the healthy/pathologic status (HC = healthy, LIC = lichen).

To investigate the existence of a disease-specific microbiota hint on the scalp of LPP patients, we evaluated the bacterial community composition at all different taxonomic levels (S1 File, sheets 4–9) and then we applied a statistical approach based on a regression linear model. As a result, three taxa at the phylum level were found as significantly different among all paired couples if considering the swab (Fig 1).

At the phylum level all other coupled layers have been tested (DE = Deep Epidermis, D = Dermis HYP = Hypodermis), but no other statistically significant result emerged. Table 1 reports all the significant results found for the other coupled pairs. Noteworthy, only the family of *Microbacteriaceae* was significantly different in the dermis layer. All the other significative results reported at the family and genus levels were found for the swab microbiota.

**Table 1:** Statistically different taxa emerged from the application of a multivariable linear regression model. To test the association between microbial taxa healthy abundances versus Lichen diagnosis, the software MaAsLin2 has been used at different taxonomic level for swab and sub-epidermal layers (swab, deep epidermis, dermis, hypodermis).

Layer	taxa level	feature	coeff	stderr	N	pval
dermis	family	Microbacteriaceae	-1.03	0.2	8	0.002
swab	phylum	Cyanobacteria	0.75	0.27	26	0.01
swab	phylum	Euryarchaeota	0.21	0.1	26	0.053
swab	phylum	Firmicutes	-0.56	0.27	26	0.051
swab	family	Pasteurellaceae	-1.02	0.39	26	0.014
swab	family	Unassigned_Lactobacillales	-0.92	0.29	26	0.004
swab	family	Neisseriaceae	-0.73	0.28	26	0.017
swab	family	Other_Firmicutes	-0.63	0.24	26	0.013
swab	family	Leptotrichiaceae	-0.61	0.25	26	0.021
swab	family	Clostridiaceae	0.1	0.04	26	0.033
swab	family	Cystobacteraceae	0.1	0.04	26	0.034
swab	family	Chlamydomonadaceae	0.1	0.04	26	0.035
swab	family	Unassigned_Rhodospirillales	0.1	0.04	26	0.037
swab	family	Unassigned_Pseudomonadales	0.11	0.05	26	0.038
swab	family	Erythrobacteraceae	0.11	0.05	26	0.039
swab	family	Phormidiaceae	0.12	0.06	26	0.041
swab	family	Unassigned_Rickettsiales	0.12	0.06	26	0.044
swab	family	Pseudanabaenaceae	0.12	0.06	26	0.047
swab	family	Aeromonadaceae	0.13	0.06	26	0.039
swab	family	Unassigned_Chroococcales	0.14	0.06	26	0.042
swab	family	Rhodospirillaceae	0.32	0.12	26	0.018
swab	family	Xanthomonadaceae	0.38	0.16	26	0.023
swab	family	Oxalobacteraceae	0.4	0.17	26	0.026
swab	family	Bradyrhizobiaceae	0.49	0.23	26	0.047
swab	family	Unassigned_Burkholderiales	0.53	0.22	26	0.023
swab	family	Caulobacteraceae	0.6	0.24	26	0.018
swab	family	Other_Cyanobacteria	0.86	0.31	26	0.01
swab	genus	Unclassified_Pasteurellaceae	-1.02	0.39	26	0.014
swab	genus	Unclassified_Neisseriaceae	-0.67	0.29	26	0.031
swab	genus	Unclassified_Leptotrichiaceae	-0.56	0.24	26	0.03
swab	genus	Verrucomicrobium	0.09	0.04	26	0.033
swab	genus	Haloferula	0.09	0.04	26	0.033
swab	genus	Clostridium	0.1	0.04	26	0.033
swab	genus	Unclassified_Cystobacteraceae	0.1	0.04	26	0.034
swab	genus	Unclassified_Erythrobacteraceae	0.1	0.04	26	0.034
swab	genus	Novosphingobium	0.1	0.04	26	0.034
swab	genus	Unclassified_Pseudanabaenaceae	0.1	0.04	26	0.036
swab	genus	Lysobacter	0.11	0.05	26	0.036
swab	genus	Unclassified_Hyphomicrobiaceae	0.11	0.05	26	0.036
swab	genus	Mucilaginibacter	0.12	0.05	26	0.037
swab	genus	Pedobacter	0.12	0.05	26	0.038
swab	genus	Pseudoxanthomonas	0.12	0.06	26	0.04
swab	genus	Unclassified_Phormidiaceae	0.12	0.06	26	0.041
swab	genus	Frondihabitans	0.12	0.06	26	0.043
swab	genus	Unclassified_mitochondria	0.12	0.06	26	0.044
swab	genus	Aeromonas	0.13	0.06	26	0.039
swab	genus	Unclassified_Chlamydomonadaceae	0.13	0.06	26	0.041
swab	genus	Caulobacter	0.15	0.06	26	0.013
swab	genus	Clostridium_2	0.2	0.07	26	0.006
swab	genus	Unclassified_Pseudomonadaceae	0.27	0.12	26	0.036
swab	genus	Unclassified_Xanthomonadaceae	0.28	0.13	26	0.049
swab	genus	Arthrobacter	0.35	0.16	26	0.044
swab	genus	Unclassified_Bradyrhizobiaceae	0.49	0.23	26	0.047
swab	genus	Other_Cyanobacteria	0.89	0.32	26	0.009

**Fig 2:**
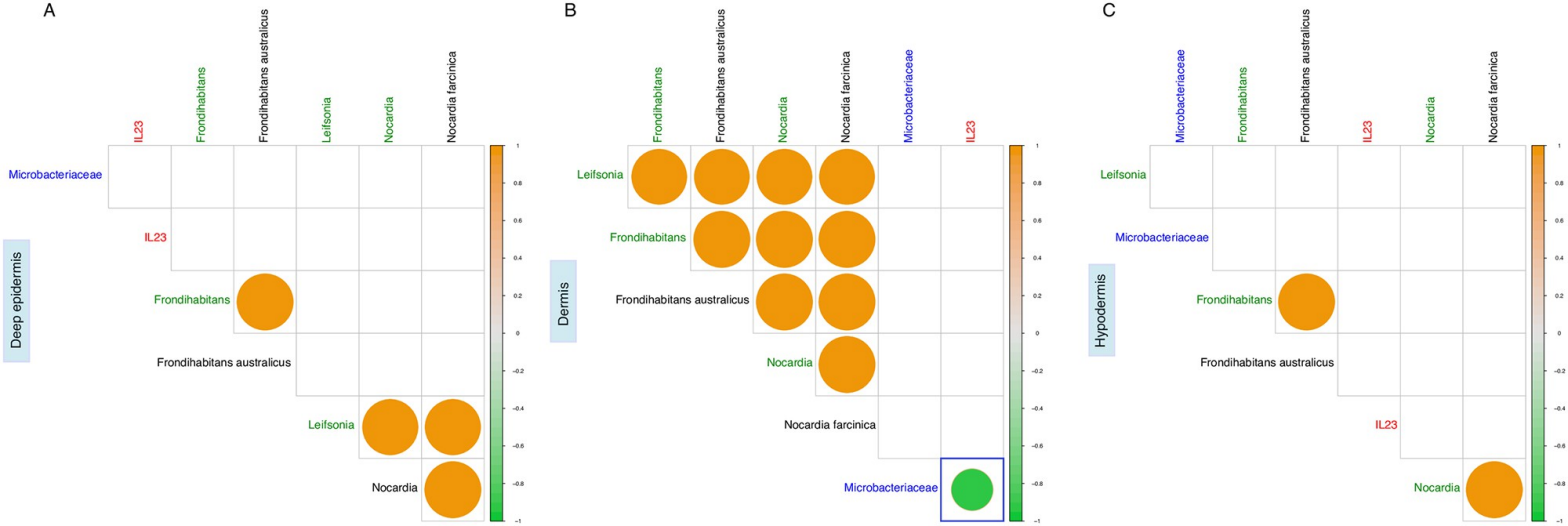
Significative correlation between taxa (genus and species) belonging to *Microbacteriaceae* family and IL-23 in the three scalp layers. Spearman correlations between families (blue), genera (green) and species (black) normalized abundances together with IL-23 ΔCt values. Panel A—deep epidermis, panel B—dermis, panel C—hypodermis. Only statistically significant correlations (p<0.05) have been plotted; the blue delimited square is relative to the only significant correlation between IL-23 and taxonomic data. The color graduated scale ranges from -1 (green—negative correlations) to 1 (orange—positive correlations).

We than investigated about a possible statistical correlation between the genus and species belonging to the *Microbacteriaceae* family (together with the family abundance itself) against the *IL-23* real time ΔCt values in each one of the three biopsy layers. Only the *Microbacteriaceae* family strongly and negatively correlated (R = -0.92, p<0.05) with the *IL-23* mRNA expression (Fig 2). Noteworthy statistical significance dissolved at genus and species levels (Fig 2B).

### Metabolic function prediction in LPP skin biopsy layers

PiCRUSt software was used to predict microbiota associated biochemical pathways in LPP and healthy samples. Pathways that significantly differed between healthy and LPP samples were selected by applying a Welch’s Test for Unequal Variances corrected by multiple tests.

In the biopsy dermis layers a total of thirty-two metabolic pathways significantly differed between healthy and LPP samples (S3 Fig and S1 Table). Differences in mean proportions statistics evidenced five pathways out of thirty-two that were downregulated in LPP samples and specifically: glycolysis and gluconeogenesis, methane metabolism and three pathways belonging to “genetic information processing” metabolism (i.e. aminoacyl-tRNA biosynthesis, RNA polymerase and ribosome).

### GC-MS VOM profiles

Complete profiles of VOMs in six LPP versus six healthy subjects were obtained by mass spectrometry and as a result a total of 73 compounds emerged (S2 Table). Only two VOMs significantly differed between the group of healthy and LPP subjects (correct p < 0.05); specifically, the 4-Terpinenol and the Ionene were detected (Table 2). Both the compounds have a higher relative mean frequency in LPP samples.

**Table 2:** VOM mean relative frequency assessed in STAMP software using a two-sided Welch’s t-test corrected by using a Benjamini-Hochberg (p < 0.05). Urine sample metabolites detected in LPP patients were compared to healthy subjects.

Compound	Healthy: mean rel.freq. (st.dev)	LPP: mean rel.freq. (st.dev)	p value	q value	Diff. between means	95.0% lower CI*	95.0% upper CI
Ionene	1.64 (0.3983)	3.28 (0.8859)	0.0072	0.0411	-1.6345	-2.6634	-0.6056
4-Terpinenol	0.63 (0.1030)	1.15 (0.2603)	0.0050	0.0393	-0.5197	-0.8202	-0.2193

*CI Confidence Interval

## 4. Discussion

Although numerous studies stated the role of bacterial populations in many skin conditions [40–43], the role of the scalp microbiome in LPP has not been investigated yet. In a previously published work, we reported preliminary data on the superficial epidermis of fifteen LPP subjects [20]. In the present study, we showed for the first time, the peculiar features of the core microbiome in cutaneous samples from LPP patients. Also, we contextually studied the differences in microbial populations inhabiting the scalp and its subepidermal compartments in subjects affected by LPP compared to those in a healthy cohort.

Increasing evidence is suggesting the role of the dysbiosis of the complex commensal communities inhabiting the human body, including the skin, on the modulation of the innate and adaptative immune response [44]. This can result in the development of autoimmune diseases with arising inflammation, including Lichen planus (LP). LPP is the form of LP affecting the scalp and hair for which Th17-derived cytokines (IL-17 and IL-23) and it has been reported to be involved in the loss of immune privilege and inflammation [45].

In the current study, we highlighted increased abundances of the phyla Cyanobacteria and Euryarcheota in LPP group compared to the healthy group. Firmicutes were found also less abundant in LPP group. Noteworthy, all these statistically significant phyla (p < 0.05) were found in the swab samples with the only exception of *Microbacteriaceae* family harboring the dermis layer. These results suggest the involvement of these bacteria in the exacerbation and severity of LPP, with a day by day raising incidence as the result of an improvement of a correct diagnosis by physicians but also linked to an increased impact of etiological factors, especially the environmental ones [13]. In 1994 the Industrial Toxicology Research Center reported for the first time the negative effect of air pollution on the scalp [14].

Nowadays we are witnessing to shifting to metro cities, especially for young people and this produces an increase of scalp discomfort and pains as results from exposure to pollution. As a consequence of pollution, particulate matter and heavy metals may accumulate on the hair [13], inducing oxidative stress by increasing the production of reactive oxygen species [46] (ROS) and leading to clinical conditions related to hair loss, including LPP [47].

Also, in the last decade some members of the Cyanobacteria phylum have been reported to bloom in the atmosphere because of the global climate change [48] but especially of air pollution [49]. This evidence could in some way correlate with the higher abundance of Cyanobacteria we found in LPP cohort. Members of this phylum and the metabolites they produced are reported to be responsible of some negative effects on human health and their extensive blooming leads to several consequences, including negative effects on the skin (irritation, rashes, desquamation, swelling, sores, allergic reactions) [50].

At the same time, it could also be hypothesized that lipopolysaccharide endotoxins which are typical components of the plasma membranes of Gram-negative microorganisms such as *Cyanobacteria* could play a role in the etiopathology of LPP by the stimulation of the immune system [51]. Therefore, *Euryarcheota* phylum was also found to be higher in LPP samples.

Even though archaeal colonization of human anatomical sites, including the skin, has long been neglected by researchers, there is a growing number of reports regarding their occurrence at sites of infection [52]. This finding is in line with the hypothesis that infection must be considered as one of the drivers triggering LPP manifestations.

Also, compared to the healthy controls, LPP samples have fewer *Firmicutes*. This is in line with previous findings on LP affecting the oral mucosa [19].

A statistically significant difference resulted in the *Microbacteriaceae* abundance (p < 0.05) at dermis layer of LPP group if compared with control samples. A shift in the *Microbacteriaceae* family abundance has been recently linked to the susceptibility to the otitis media in outer ear skin due to SPINK5 gene variants [53].

We also found a higher microbial diversity in LPP group; this is in line with previous findings [54] where a higher susceptibility of an unhealthy scalp leads the ground to an easier colonization of microorganisms.

Starting with 16S sequencing data, we also explored the differences in predicted KEGG pathways between LPP and healthy samples.

Glycolysis and gluconeogenesis, methane metabolism and three pathways belonging to genetic information processing were found to be downregulated in LPP samples.

Increase in methane metabolism has been reported in atopic dermatitis (AD) [55], another dermatologic disease strongly associated with unbalanced immunological response. It has been hypothesized that dietary metabolites, including methane, produced by gut microbiota, may impact on host immune system leading to an increase in susceptibility to the disease, including LPP.

Hair follicle (HF) possess interesting features related to carbohydrate metabolism including aerobic glycolysis, storage and mobilization of glycogen and the amount of ATP available for follicle activity resulted as greatly influenced by the relative contributions of these metabolic pathways [56]. Because of their alteration, also the hair growth may be altered.

More recently, the role of glycogen metabolism in human HF (HF) has been investigated by Figlak and collaborators [57] and HF outer root sheath (ORS) were found to be the main responsible of glycogen’s synthesis with high levels of glycogen found in the ORS of anagen HF and decreased levels in catagen and absent in telogen phase of the hair cycle. The downregulation of glycolysis and gluconeogenesis metabolism that we found in LPP samples is in line with the above assumptions. Therefore, also peroxisome proliferator-activated receptor *γ* (PPAR-*γ*) has been reported to play a role in the pathogenesis of cicatricial alopecia, including LPP [58]. This ligand-activated nuclear receptor is linked to lipid homeostasis but also to inflammatory regulation of sebaceous glands whose function is critical for hair follicle cycling. The main function of sebaceous gland is lipogenesis and glycogen and glycerophosphate represent the main substrates for the synthesis of sebum lipids [57].

Pathways related to aminoacyl-tRNA biosynthesis, RNA polymerase and ribosome were downregulated in LPP samples. Certain aminoacyl-tRNA synthetases (ARSs) are reported to be closely related to different types of immune responses since their involvement in the maturation, transcription, activation and recruitment of immune cells [59]. Interestingly, they can also act as regulators and signaling molecules in various immune diseases including LPP. Indeed, in LPP the immune privilege collapse of the hair follicle’s epithelial stem cell niche is observed [60].

In LPP sample, the downregulation of pathways related to the immune response corresponds to a significant increase in cellular antigens pathway. This is in line with previous evidence on the role of microbial-derived antigens for the susceptibility of the HF under an autoimmune attack [61]. Therefore, associated autoantigen has been recently identified for Lichen Planus [62].

We contextually detected a significant up-regulation in the production of ribosomes and RNA polymerase activity which may be linked to cellular stress, a well know reported marker of LP. Indeed, Wang and collaborators reported a dysregulation of ribosomes biosynthesis as a consequence of cellular stress [48].

Some other pathways, mainly related to metabolism of glycosaminoglycan (GAG) degradation, isoquinoline alkaloid biosynthesis, lipopolysaccharides biosynthesis and antimicrobial resistance, bacterial chemotaxis and flagellar assembly were detected with higher normalized abundance values and resulted predominant in LPP samples.

The findings of the present work are in line with our previous investigation on another form of auto-immune disease affecting the scalp, Alopecia areata [63]. Indeed, the alterations of GAG degradation have been reported as responsible of abnormalities in hair morphology [39] and of the alteration of the morphology of the connective tissue surrounding the HF [64]. Bacteria themselves can use GAGs, as a mask to avoid the recognition by the immune system collaborators [65].

Some recent evidence argued about the possibility that significant differences in OTU abundances may be limited to the epidermal communities, and possibly no effect on the communities inhabiting subepidermal compartments [66]. Accordingly, the great majority of statistically significant taxa at genus and family levels emerged when we compared swab samples.

Some specific upregulated pathways in the LPP sample, such as bacterial chemotaxis, flagellar assembly, and lipopolysaccharide biosynthesis, could be an indication of the presence of specific bacterial community, probably inhabiting the dermal compartment.

Therefore, bacterial chemotaxis is reported to promote activated T lymphocytes in autoimmune disorders [66, 67] and to ensure the access of bacterial species to host niches, where proapoptotic host cell factors were delivered [67].

The pathway relative to isoquinoline alkaloids biosynthesis was predicted to be significantly higher in LPP samples. The synthesis of this type of alkaloids, produced as secondary metabolites by several microorganisms, may be a consequence of the enrichment of microbial population by members of Cyanbacteria.

Skin microbiota, both superficial and the deeper one, comes into contact with epidermal cells; keratinocytes interact also with the cutaneous immune system [68] influencing the immunological response of the scalp and HF [69]. In the present work we noticed in the scalp a strong negative correlation between the *Microbacteriaceae* family and the *IL-23* mRNA levels. The IL-23/IL-17 axis has a pivotal role in the pathogenesis of many chronic inflammatory diseases, including LP [70]. One proposed mechanism from LPP regards the T cell-mediated destruction of the hair follicle bulge and IL-12 and IL-23 are key cytokines involved in this process [71].

By correlating the gene expression of one of the main cytokines involved in the T cell-mediated response in subjects affected by LPP and the microbial population inhabiting the scalp of these subjects, we highlighted the strict connection existing between microbial dysbiosis and clinical manifestations of LPP.

Moreover, by inspecting the urine metabolomic sample profiles we identified two VOMs that resulted significantly higher in LPP than healthy samples. Ionene belongs to a family of polycations known for their antimicrobial activity [72] and probably its higher detected presence in LPP samples may derive from the food or topical medication usage. No scientific association emerged for autoimmune or inflammatory disease and ionene. On the other hand, low concentration of the 4-Terpineol exerts a broad-spectrum antimicrobial and anti-inflammatory effect; this monoterpen is used in different topical formulations based on essential oils [73]. Specifically, the anti-inflammatory effect relies on a selective regulation of monocyte activity, thus toning down immune responses in the skin by inhibiting the production of IL-1β, IL-6 and IL-10 [74]. In another study *in vitro* tests reveal how only terpinen-4-ol suppressed the production after 40 h of TNFα, IL-1β, IL-8, IL-10 and PGE2 by LPS-activated monocytes [75]. 4-Terpineol and alpha-terpineol have been used in skin topical formulation, but the former has usually a higher percentage than the latter. In our metabolomics results we detected both the compounds but the alpha-terpineol resulted as not statistically significant (S2 Table). As confirmed by our data, the LPP chronic inflammatory disease has an immune response that is triggered by pro inflammatory cytokine IL-23, whose activity together with its downstream effector molecules have been considered as therapeutic targets.

The involvement of the microbiota, both as population and pathways, open to novel therapeutic approaches for the treatment of LPP. Currently available therapeutic options mainly aim to counteract the progression of the lesion with systemic oral corticosteroid, griseofulvin, dapsone, and thalidomide therapy as the first line therapy in case of severe, quickly evolutive LPP [76]. Tosti and collaborators reported the efficacy of clobetasol propionate foam 0.05% [77].

As suggested for atopic dermatitis [78], another inflammatory cutaneous disease, it can be postulated that the use of corticosteroid might suffice to normalize the cutaneous microbial communities on the scalp of LPP subjects. Although the topical treatment with high-potency corticosteroids remains the most effective and safest therapy in the early treatment of LPP, the findings of the present work shed new light of the use of postbiotics [79]. The term “postbiotic” refers to bioactive compounds produced as a result of the metabolic activity of probiotic bacteria. Due to their high specificity of action on resident microbiota and their health-promoting effects in dermatological conditions, postbiotics represent a novel frontier in dermatology.

## 5. Conclusions

In conclusions, by investigating the bacterial communities inhabiting the superficial epidermis and three biopsy sub-cutaneous layers and reconstructing the biochemical pathways, we shed lights on LPP, a neglected skin pathology. Our results indicated that bacterial community composition and the relative richness are subject to dramatic alterations in the scalp of LPP patients. Thanks to a systematic and multi-level analysis we described the differential abundance of various taxa in the swab and dermis of LPP patients together with their related biochemical pathways. Metagenomics approaches and multi-omics studies are needed to further identify bacterial species and pathways related to LPP immune-related functions.

As the main limitation of the study was the small sample size. Further studies consisting of larger number of patients are needed to better support the provided evidence.

## Supporting information

S1 Table Picrust significant pathway predictions between healthy and LPP dermis samples. Thirty-two PiCRUSt metabolic pathways from dermis layer belonging to healthy and LPP samples. Statistics are based on Welch’s test for group comparison and Benjamini-Hochberg procedure for multiple test correction. Rel.freq = relative frequency; std.dev. = standard deviation; CI = confidence interval.
(DOCX)

S2 Table Welch test comparison of volatile organic metabolites (VOMs) in LPP versus healthy samples. (DOCX)

S1 Fig IL23 expression in control vs. LPP sub-epidermal layers. mRNA levels of IL-23 detected by RT-PCR plus error bars.
(DOCX)

S2 Fig Alpha diversity metrics boxplots. Three different alpha diversity metrics have been computed in Qiime2. PanelA: Observed OTUs; PanelB: Shannon’s diversity index. the dermis layer belonging to healthy and LPP samples PanelC: Faith’s Phylogenetic Diversity (phylogenetic generalization of species richness).
(DOCX)

S3 Fig Statistically significant differences in biochemical pathways harbouring LPP versus healthy samples. Pathway normalized abundances from PICRUSt have been analyzed by Welch’s test than corrected by multiple test (Benjamini-Hochberg). Only statistically significant pathways (q value < 0.05) have been reported. Higher mean proportions for healthy and LPP subjects have been plotted as orange or blus dots, respectively. Biochemical pathways which differ significantly in relative abundance between the dermis samples of healthy and LPP samples. The statistical analysis was performed and visualized using the STAMP package. Mean abundance (mean proportion) and difference in mean proportion for pathways showing significant difference in abundance are shown. The 95% confidence intervals and statistical significance (corrected q value) are indicated as well.
(DOCX)

S1 File Qiime2 and plugin outputs. Sheet 1. “lichen metadata”: Sample ID, layer and type of sampled material (biopsy or swab); sheet 2. “demux-filtered-stats”: Qiime2 quality read control demultiplex statistics; sheet 3. “deblur_stats”: Denoising statistics from QIIME2 Deblur plugin; sheet 4. “phylum_assigned_OTU”: Qiime 2 phylum relative frequency table; sheet 5. “class_assigned_OTU”: Qiime 2 class relative frequency table; sheet 6. “order_assigned_OTU”: Qiime 2 order relative frequency table; sheet 7. “family_assigned_OTU”: Qiime 2 family relative frequency table; sheet 8. “genus_assigned_OTU”: Qiime 2 genus relative frequency table; sheet 9. “species_assigned_OTU”: Qiime 2 species relative frequency table.
(XLSX)
