# Supplementary material for: Lichen Planopilaris: The first biopsy layer microbiota inspection
Source: PLoS One. 2022 Jul 18;17(7):e0269933. doi: 10.1371/journal.pone.0269933 (PMC9292073; doi:10.1371/journal.pone.0269933)
Supplement: S1 Table — Thirty-two PiCRUSt metabolic pathways from dermis layer belonging to healthy and LPP samples. Statistics are based on Welch’s test for group comparison and Benjamini-Hochberg procedure for multiple test correction. Rel.freq = relative frequency; std.dev. = standard deviation; CI = confidence interval. (DOCX) [file pone.0269933.s001.docx]

# Supplementary Table S1: Picrust significant pathway predictions between healthy and LPP dermis samples. Thirty-two PiCRUSt metabolic pathways from dermis layer belonging to healthy and LPP samples. Statistics are based on Welch’s test for group comparison and Benjamini-Hochberg procedure for multiple test correction. Rel.freq = relative frequency; std.dev. = standard deviation; CI = confidence interval.

| Pathway | healthy Derma: mean rel.freq.(%) | healthy Derma: std.dev.(%) | LPP Derma: mean rel.freq.(%) | LPP Derma: std.dev.(%) | p-values | q-values | Diff. betw. means | 95.0% lower CI | 95.0% upper CI |
| --- | --- | --- | --- | --- | --- | --- | --- | --- | --- |
| Unclassified;Cellular Processes and Signaling;Pores ion channels | 0.349 | 0.023 | 0.583 | 0.072 | 0.000 | 0.000 | -0.235 | -0.290 | -0.180 |
| Metabolism;Biosynthesis of Other Secondary Metabolites;Isoquinoline alkaloid biosynthesis | 0.029 | 0.004 | 0.058 | 0.014 | 0.000 | 0.005 | -0.029 | -0.039 | -0.019 |
| Unclassified;Cellular Processes and Signaling;Other transporters | 0.205 | 0.007 | 0.253 | 0.025 | 0.000 | 0.007 | -0.049 | -0.067 | -0.030 |
| Metabolism;Glycan Biosynthesis and Metabolism;Glycosphingolipid biosynthesis - ganglio series | 0.011 | 0.003 | 0.025 | 0.006 | 0.000 | 0.010 | -0.015 | -0.020 | -0.009 |
| Cellular Processes;Transport and Catabolism;Lysosome | 0.030 | 0.004 | 0.065 | 0.021 | 0.000 | 0.011 | -0.035 | -0.050 | -0.021 |
| Organismal Systems;Digestive System;Protein digestion and absorption | 0.005 | 0.001 | 0.012 | 0.003 | 0.000 | 0.011 | -0.007 | -0.009 | -0.004 |
| Unclassified;Cellular Processes and Signaling;Membrane and intracellular structural molecules | 0.464 | 0.059 | 0.748 | 0.087 | 0.000 | 0.014 | -0.283 | -0.386 | -0.181 |
| Metabolism;Biosynthesis of Other Secondary Metabolites;beta-Lactam resistance | 0.020 | 0.005 | 0.044 | 0.013 | 0.000 | 0.017 | -0.024 | -0.035 | -0.013 |
| Metabolism;Glycan Biosynthesis and Metabolism;Other glycan degradation | 0.066 | 0.013 | 0.123 | 0.034 | 0.001 | 0.018 | -0.057 | -0.084 | -0.029 |
| Unclassified;Cellular Processes and Signaling;Cell motility and secretion | 0.158 | 0.028 | 0.272 | 0.051 | 0.001 | 0.018 | -0.114 | -0.165 | -0.063 |
| Unclassified;Metabolism;Glycan biosynthesis and metabolism | 0.033 | 0.008 | 0.063 | 0.015 | 0.001 | 0.019 | -0.029 | -0.043 | -0.015 |
| Organismal Systems;Environmental Adaptation;Plant-pathogen interaction | 0.106 | 0.004 | 0.126 | 0.014 | 0.001 | 0.019 | -0.020 | -0.030 | -0.010 |
| Metabolism;Glycan Biosynthesis and Metabolism;Glycosaminoglycan degradation | 0.020 | 0.005 | 0.040 | 0.012 | 0.001 | 0.019 | -0.020 | -0.030 | -0.011 |
| Metabolism;Metabolism of Other Amino Acids;Glutathione metabolism | 0.323 | 0.026 | 0.436 | 0.068 | 0.001 | 0.020 | -0.112 | -0.167 | -0.058 |
| Environmental Information Processing;Signaling Molecules and Interaction;Cellular antigens | 0.037 | 0.009 | 0.070 | 0.018 | 0.001 | 0.022 | -0.033 | -0.050 | -0.017 |
| Metabolism;Glycan Biosynthesis and Metabolism;Lipopolysaccharide biosynthesis proteins | 0.273 | 0.051 | 0.476 | 0.064 | 0.001 | 0.023 | -0.203 | -0.289 | -0.117 |
| Environmental Information Processing;Membrane Transport;Secretion system | 1.409 | 0.098 | 1.752 | 0.177 | 0.002 | 0.024 | -0.343 | -0.519 | -0.166 |
| Cellular Processes;Cell Motility;Bacterial motility proteins | 0.678 | 0.186 | 1.342 | 0.431 | 0.002 | 0.024 | -0.664 | -1.033 | -0.295 |
| Cellular Processes;Cell Motility;Flagellar assembly | 0.230 | 0.078 | 0.507 | 0.175 | 0.002 | 0.024 | -0.276 | -0.429 | -0.124 |
| Metabolism;Biosynthesis of Other Secondary Metabolites;Penicillin and cephalosporin biosynthesis | 0.033 | 0.008 | 0.060 | 0.016 | 0.002 | 0.024 | -0.026 | -0.041 | -0.012 |
| Genetic Information Processing;Translation;Aminoacyl-tRNA biosynthesis | 1.155 | 0.025 | 1.021 | 0.099 | 0.001 | 0.024 | 0.134 | 0.063 | 0.205 |
| Cellular Processes;Cell Motility;Bacterial chemotaxis | 0.233 | 0.069 | 0.484 | 0.157 | 0.002 | 0.025 | -0.251 | -0.387 | -0.116 |
| Metabolism;Energy Metabolism;Methane metabolism | 1.016 | 0.017 | 0.943 | 0.055 | 0.002 | 0.025 | 0.073 | 0.032 | 0.115 |
| Unclassified;Metabolism;Biosynthesis and biodegradation of secondary metabolites | 0.047 | 0.010 | 0.082 | 0.019 | 0.002 | 0.025 | -0.035 | -0.054 | -0.017 |
| Environmental Information Processing;Signal Transduction;Two-component system | 1.661 | 0.098 | 2.008 | 0.196 | 0.002 | 0.026 | -0.347 | -0.530 | -0.164 |
| Metabolism;Glycan Biosynthesis and Metabolism;Lipopolysaccharide biosynthesis | 0.183 | 0.044 | 0.350 | 0.048 | 0.002 | 0.026 | -0.167 | -0.241 | -0.093 |
| Unclassified;Poorly Characterized;Function unknown | 1.462 | 0.081 | 1.770 | 0.103 | 0.002 | 0.027 | -0.308 | -0.446 | -0.170 |
| Genetic Information Processing;Transcription;RNA polymerase | 0.176 | 0.005 | 0.149 | 0.024 | 0.003 | 0.036 | 0.028 | 0.011 | 0.045 |
| Genetic Information Processing;Translation;Ribosome | 2.240 | 0.064 | 1.942 | 0.257 | 0.004 | 0.038 | 0.298 | 0.114 | 0.482 |
| Metabolism;Carbohydrate Metabolism;Glycolysis / Gluconeogenesis | 1.255 | 0.056 | 1.087 | 0.108 | 0.005 | 0.046 | 0.168 | 0.065 | 0.271 |
| Organismal Systems;Endocrine System;Renin-angiotensin system | 0.001 | 0.001 | 0.006 | 0.004 | 0.005 | 0.047 | -0.005 | -0.008 | -0.002 |
| Genetic Information Processing;Folding, Sorting and Degradation;Chaperones and folding catalysts | 0.876 | 0.040 | 0.991 | 0.061 | 0.005 | 0.047 | -0.116 | -0.184 | -0.047 |
