# Supplementary material for: Lichen Planopilaris: The first biopsy layer microbiota inspection
Source: PLoS One. 2022 Jul 18;17(7):e0269933. doi: 10.1371/journal.pone.0269933 (PMC9292073; doi:10.1371/journal.pone.0269933)
Supplement: S2 Table — (DOCX) [file pone.0269933.s002.docx]

**Supplementary Table S2:** Welch test comparison of volatile organic metabolites (VOMs) in LPP versus healthy samples.

| COMPOUNDS | Healthy: mean rel. freq. (%) | Healthy: std. dev. (%) | LPP: mean rel. freq. (%) | LPP: std. dev. (%) | p-values | q-values | Difference between means | 95.0% lower CI | 95.0% upper CI |
| --- | --- | --- | --- | --- | --- | --- | --- | --- | --- |
| (-)-carvone | 0.9066 | 0.5494 | 1.4519 | 2.1151 | 0.5982 | 0.8973 | -0.5452 | -2.9705 | 1.8801 |
| .alpha.-terpineol | 0.8283 | 0.5837 | 0.1083 | 0.1577 | 0.0691 | 0.5635 | 0.7199 | 0.0505 | 1.3894 |
| .gamma.-terpinene | 0.2550 | 0.1731 | 0.2866 | 0.3914 | 0.8735 | 0.9114 | -0.0316 | -0.4858 | 0.4225 |
| 1,1,5-trimethyl-1,2-dihydronaphthalene | 1.1549 | 0.3262 | 2.0451 | 0.8074 | 0.0584 | 0.6009 | -0.8902 | -1.8228 | 0.0423 |
| 1,5,5-trimethyl-6-methylene-cyclohexene | 0.0633 | 0.0697 | 0.3200 | 0.3922 | 0.2059 | 0.8234 | -0.2567 | -0.7066 | 0.1932 |
| 1-hexanol, 2-ethyl- | 0.1850 | 0.2686 | 0.0000 | 0.0000 | 0.1841 | 0.7798 | 0.1850 | -0.1237 | 0.4937 |
| 1-oxaspiro[4.5]dec-6-ene, 2,6,10,10-tetramethyl- | 0.2316 | 0.2146 | 0.1967 | 0.1422 | 0.7685 | 0.8783 | 0.0350 | -0.2269 | 0.2969 |
| 2(1h)-naphthalenone, 3,4,4a,5,6,7-hexahydro-1,1,4,-trimethyl | 7.6128 | 3.8508 | 8.0636 | 2.1479 | 0.8250 | 0.8866 | -0.4508 | -5.0146 | 4.1130 |
| 2,4-dimethylfuran | 0.0917 | 0.2050 | 0.1517 | 0.1645 | 0.6212 | 0.8770 | -0.0600 | -0.3235 | 0.2035 |
| 2,5-cyclohexadiene-1,4-dione, 2,6-bis(1,1,dimethylethyl) | 0.1867 | 0.1089 | 0.1317 | 0.1675 | 0.5542 | 0.9068 | 0.0550 | -0.1486 | 0.2586 |
| 2-butanone | 0.2867 | 0.1066 | 0.2300 | 0.0640 | 0.3374 | 0.7836 | 0.0567 | -0.0710 | 0.1843 |
| 2-buten-1-one, 1-(2,6,6-trimethyl-1,3-cyclohexadien-1-yl) | 1.3266 | 0.3419 | 1.8151 | 0.2967 | 0.0769 | 0.6651 | -0.4885 | -0.9408 | -0.0362 |
| 2-furanmethanol, 5-ethenyltetrahydro-a,a,trimethyl-cis | 3.7430 | 2.6270 | 1.0717 | 0.8762 | 0.0736 | 0.5892 | 2.6713 | -0.3472 | 5.6898 |
| 2-heptanone | 0.3150 | 0.2367 | 0.3683 | 0.3301 | 0.7755 | 0.8725 | -0.0534 | -0.4639 | 0.3571 |
| 2-methoxy-4-vinylphenol | 4.3400 | 4.1632 | 2.6719 | 1.8866 | 0.4415 | 0.8829 | 1.6681 | -3.1696 | 6.5058 |
| 2-methoxythiophene | 0.7849 | 0.4681 | 1.3968 | 0.9007 | 0.2169 | 0.8219 | -0.6119 | -1.6705 | 0.4468 |
| 2-pentanone | 2.8649 | 1.2207 | 1.3216 | 1.0495 | 0.0583 | 0.6993 | 1.5433 | -0.0657 | 3.1523 |
| 2h-1-benzopyran, 3,4,4a,5,6,8a-hexahydro-2,5,5,8a-tetramethyl- | 0.2317 | 0.1227 | 0.2483 | 0.0437 | 0.7838 | 0.8682 | -0.0167 | -0.1578 | 0.1245 |
| 2h-pyran, 2-ethenyltetrahydro-2,6,6,-trimethyl | 0.1267 | 0.0999 | 0.0617 | 0.0910 | 0.3077 | 0.8521 | 0.0650 | -0.0698 | 0.1998 |
| 3-cyclohexene-1-methanol-2-hydroxy-alpha,alpha,4-trimethyl | 1.2300 | 1.7747 | 0.3484 | 0.1761 | 0.3184 | 0.7905 | 0.8816 | -1.1567 | 2.9199 |
| 3-hexanone | 0.9866 | 0.1823 | 0.6516 | 0.4398 | 0.1617 | 0.7764 | 0.3350 | -0.1736 | 0.8435 |
| 3-hexanone, 4-methyl- | 0.1433 | 0.1190 | 0.0300 | 0.0671 | 0.1011 | 0.6069 | 0.1133 | -0.0279 | 0.2545 |
| 3-hexanone, 5-methyl- | 0.1317 | 0.1342 | 0.0383 | 0.0857 | 0.2243 | 0.7690 | 0.0933 | -0.0692 | 0.2559 |
| 4-(2,4,4-trimethyl-cyclohexa-1,5-dienyl)-but-3-en-2-one) | 1.2633 | 0.5823 | 1.2951 | 0.5689 | 0.9320 | 0.9451 | -0.0319 | -0.8431 | 0.7794 |
| 4-(2,6,6-trimethylcyclohexa-1,3-dimenyl)but-3-en-2-one) | 1.2632 | 0.5259 | 1.9235 | 0.6381 | 0.1056 | 0.5848 | -0.6603 | -1.4883 | 0.1678 |
| 4-heptanone | 18.4485 | 5.6228 | 15.3677 | 3.5123 | 0.3278 | 0.7867 | 3.0808 | -3.7017 | 9.8633 |
| Acetic acid | 0.4083 | 0.3541 | 0.5884 | 0.4514 | 0.4997 | 0.8995 | -0.1800 | -0.7561 | 0.3960 |
| Acetone | 1.4366 | 0.4317 | 1.1267 | 0.3040 | 0.2219 | 0.7988 | 0.3099 | -0.2244 | 0.8442 |
| Benzaldehyde | 1.7049 | 2.2441 | 1.0968 | 1.3333 | 0.6163 | 0.8874 | 0.6081 | -2.0758 | 3.2921 |
| Benzaldehyde, 2,4-dimethyl- | 0.2783 | 0.2167 | 0.9384 | 0.6850 | 0.0858 | 0.6178 | -0.6601 | -1.4466 | 0.1265 |
| Benzene, 1,2,4,5-tetramethyl- | 0.2616 | 0.2510 | 1.9133 | 3.4937 | 0.3395 | 0.7639 | -1.6516 | -5.6660 | 2.3627 |
| Benzene, 1-ethenyl-4-methoxy- | 0.6800 | 0.5027 | 0.2117 | 0.1439 | 0.0936 | 0.6126 | 0.4683 | -0.1083 | 1.0449 |
| Benzene, 1-methyl-4-(1-methylenyl) | 6.8763 | 4.3718 | 5.6855 | 1.9447 | 0.5954 | 0.9122 | 1.1908 | -3.8833 | 6.2650 |
| Benzene, 4-ethenyl-1,2,-dimethoxy | 0.5133 | 0.3624 | 0.5967 | 0.1680 | 0.6547 | 0.8570 | -0.0834 | -0.5051 | 0.3383 |
| Benzyl alcohol, .alpha.-isobutyl-2,4,6-trimethyl | 0.1867 | 0.2221 | 0.2483 | 0.1529 | 0.6216 | 0.8606 | -0.0617 | -0.3351 | 0.2117 |
| Butanoic acid | 0.1233 | 0.1322 | 1.1485 | 1.6781 | 0.2307 | 0.7223 | -1.0251 | -2.9531 | 0.9029 |
| Butanoic acid, 2-methyl- | 0.3716 | 0.2459 | 0.4817 | 0.4817 | 0.6621 | 0.8513 | -0.1101 | -0.6752 | 0.4551 |
| Butanoic acid, 3-methyl- | 0.2133 | 0.2418 | 0.4167 | 0.5346 | 0.4638 | 0.9025 | -0.2034 | -0.8244 | 0.4177 |
| Cyclohexene, 1-methyl-4-(1-methylethylidene) | 0.3117 | 0.3753 | 0.5299 | 0.6146 | 0.5164 | 0.8853 | -0.2183 | -0.9567 | 0.5201 |
| Cyclohexene, 3-methyl-6-(1-met | 0.1900 | 0.2361 | 0.1400 | 0.1131 | 0.6818 | 0.8613 | 0.0500 | -0.2254 | 0.3254 |
| D-limonene | 0.3600 | 0.2928 | 0.2500 | 0.1588 | 0.4821 | 0.9134 | 0.1100 | -0.2357 | 0.4557 |
| Dimethyl trisulfide | 0.6816 | 0.2410 | 0.0000 | 0.0000 | 0.0615 | 0.1050 | 0.6816 | 0.4045 | 0.9587 |
| Disulfide, dimethyl | 1.5066 | 0.9008 | 1.0950 | 0.5468 | 0.4072 | 0.8376 | 0.4116 | -0.6696 | 1.4927 |
| Ethyl acetate | 2.4515 | 0.7742 | 1.3450 | 1.7440 | 0.2365 | 0.7094 | 1.1064 | -0.9175 | 3.1304 |
| Ethylcyclopentanone | 0.1050 | 0.0618 | 0.1283 | 0.1133 | 0.6970 | 0.8506 | -0.0233 | -0.1573 | 0.1106 |
| Falcarinol | 0.0717 | 0.0552 | 0.0633 | 0.0650 | 0.8317 | 0.8807 | 0.0083 | -0.0769 | 0.0936 |
| Furan, 2,3,5-trimethyl- | 0.2983 | 0.1979 | 0.4667 | 0.7309 | 0.6375 | 0.8661 | -0.1684 | -1.0066 | 0.6698 |
| Furan, 2,5-dimethyl- | 0.8516 | 0.6375 | 0.6083 | 0.4656 | 0.5078 | 0.8917 | 0.2433 | -0.5533 | 1.0399 |
| Furan, 2-ethyl-5-methyl | 1.3782 | 1.0210 | 1.6233 | 1.3744 | 0.7561 | 0.8924 | -0.2451 | -1.9706 | 1.4805 |
| Furan, 2-methyl- | 0.2416 | 0.1764 | 0.1383 | 0.1298 | 0.3184 | 0.8186 | 0.1033 | -0.1175 | 0.3242 |
| Furan, tetrahydro-2,2-dimethyl | 0.2183 | 0.0908 | 0.1250 | 0.1121 | 0.1799 | 0.8096 | 0.0933 | -0.0513 | 0.2379 |
| Heptanoic acid | 0.7000 | 0.4954 | 0.8150 | 0.1993 | 0.6455 | 0.8607 | -0.1151 | -0.6872 | 0.4571 |
| Hexanal | 2.9414 | 0.9568 | 3.5787 | 2.1687 | 0.5670 | 0.9072 | -0.6374 | -3.1533 | 1.8786 |
| Hexanoic acid | 0.8633 | 0.3211 | 0.8884 | 0.2063 | 0.8865 | 0.9118 | -0.0251 | -0.4146 | 0.3644 |
| Isomaltol | 0.1283 | 0.1540 | 0.1617 | 0.1783 | 0.7584 | 0.8807 | -0.0333 | -0.2688 | 0.2021 |
| Methanethiol | 1.7799 | 0.7165 | 2.0901 | 0.7753 | 0.5261 | 0.8809 | -0.3102 | -1.3630 | 0.7426 |
| N-decanoic acid | 0.4566 | 0.1539 | 0.3600 | 0.1937 | 0.4041 | 0.8557 | 0.0966 | -0.1517 | 0.3449 |
| Ionene | 1.6449 | 0.3984 | 3.2769 | 0.8859 | 0.0073 | 0.0411 | -1.6345 | -2.6634 | -0.6056 |
| Naphthalene | 0.1750 | 0.1602 | 0.1667 | 0.1547 | 0.9350 | 0.9350 | 0.0083 | -0.2136 | 0.2303 |
| Naphthalene, 1,6-dimethyl-4 | 0.4517 | 0.3572 | 0.3250 | 0.3373 | 0.5771 | 0.9033 | 0.1266 | -0.3631 | 0.6164 |
| Nonanal | 1.1816 | 0.3044 | 1.3134 | 0.2717 | 0.4869 | 0.8988 | -0.1318 | -0.5391 | 0.2755 |
| Nonanoic acid | 4.3844 | 2.6163 | 4.7955 | 2.6100 | 0.8086 | 0.8821 | -0.4111 | -4.0936 | 3.2714 |
| Octanal | 0.2216 | 0.3415 | 0.1350 | 0.0983 | 0.6060 | 0.8905 | 0.0866 | -0.3052 | 0.4784 |
| Octanoic acid | 1.0233 | 0.2889 | 1.3868 | 0.6218 | 0.2742 | 0.7896 | -0.3635 | -1.0872 | 0.3602 |
| P-cresol | 5.3547 | 1.9293 | 8.5815 | 6.1863 | 0.3084 | 0.8223 | -3.2269 | -10.3286 | 3.8749 |
| P-cymene | 3.7564 | 3.3074 | 2.3534 | 0.9998 | 0.3994 | 0.8715 | 1.4030 | -2.3926 | 5.1986 |
| Phenol | 0.7433 | 0.3882 | 0.6800 | 0.1545 | 0.7456 | 0.8947 | 0.0633 | -0.3849 | 0.5114 |
| Phenol, 2,4-bis(1,1-dimethylethyl) | 2.1299 | 1.3479 | 4.1987 | 1.7127 | 0.0613 | 0.5514 | -2.0688 | -4.2568 | 0.1193 |
| Phenol, 2-methoxy- | 0.3083 | 0.1449 | 0.4950 | 0.2822 | 0.2272 | 0.7437 | -0.1867 | -0.5180 | 0.1446 |
| Propanoic acid, 2-methyl-, 1-(1,1,-dimethylethyl)-2-methyl-1,3-propanedyl ester | 0.0900 | 0.1086 | 0.2217 | 0.1399 | 0.1294 | 0.6653 | -0.1317 | -0.3097 | 0.0463 |
| Terpinen-4-ol | 0.6283 | 0.1030 | 1.1517 | 0.2603 | 0.0051 | 0.0393 | -0.5197 | -0.8202 | -0.2193 |
| Tetracyclo[6.1.0.0(2.4)0(5,7) nonane 3,6,9-trimethyl- | 0.3150 | 0.3548 | 0.4434 | 0.5937 | 0.6887 | 0.8549 | -0.1284 | -0.8391 | 0.5823 |
