# Supplementary material for: Lichen Planopilaris: The first biopsy layer microbiota inspection
Source: PLoS One. 2022 Jul 18;17(7):e0269933. doi: 10.1371/journal.pone.0269933 (PMC9292073; doi:10.1371/journal.pone.0269933)
Supplement: S1 Fig — mRNA levels of IL-23 detected by RT-PCR plus error bars. (DOCX) [file pone.0269933.s003.docx]

**Supplementary Figure 1.** IL23 expression in control vs. LPP sub-epidermal layers. mRNA levels of IL-23 detected by RT-PCR plus error bars.
