# Supplementary material for: Lichen Planopilaris: The first biopsy layer microbiota inspection
Source: PLoS One. 2022 Jul 18;17(7):e0269933. doi: 10.1371/journal.pone.0269933 (PMC9292073; doi:10.1371/journal.pone.0269933)
Supplement: S2 Fig — Three different alpha diversity metrics have been computed in Qiime2. PanelA: Observed OTUs; PanelB: Shannon’s diversity index. the dermis layer belonging to healthy and LPP samples PanelC: Faith’s Phylogenetic Diversity (phylogenetic generalization of species richness). (DOCX) [file pone.0269933.s004.docx]

**Supplementary Figure 2.** Alpha diversity metrics boxplots. Three different alpha diversity metrics have been computed in Qiime2. PanelA: Observed OTUs; PanelB: Shannon’s diversity index. the dermis layer belonging to healthy and LPP samples PanelC: Faith’s Phylogenetic Diversity (phylogenetic generalization of species richness).

#
