# Supplementary material for: Lichen Planopilaris: The first biopsy layer microbiota inspection
Source: PLoS One. 2022 Jul 18;17(7):e0269933. doi: 10.1371/journal.pone.0269933 (PMC9292073; doi:10.1371/journal.pone.0269933)

# Supplementary Figure 3. Statistically significant differences in biochemical pathways harbouring LPP versus healthy samples. Pathway normalized abundances from PICRUSt have been analyzed by Welch’s test than corrected by multiple test (Benjamini-Hochberg). Only statistically significant pathways (q value < 0.05) have been reported. Higher mean proportions for healthy and LPP subjects have been plotted as orange or blus dots, respectively. Biochemical pathways which differ significantly in relative abundance between the dermis samples of healthy and LPP samples. The statistical analysis was performed and visualized using the STAMP package. Mean abundance (mean proportion) and difference in mean proportion for pathways showing significant difference in abundance are shown. The 95% confidence intervals and statistical significance (corrected q value) are indicated as well.


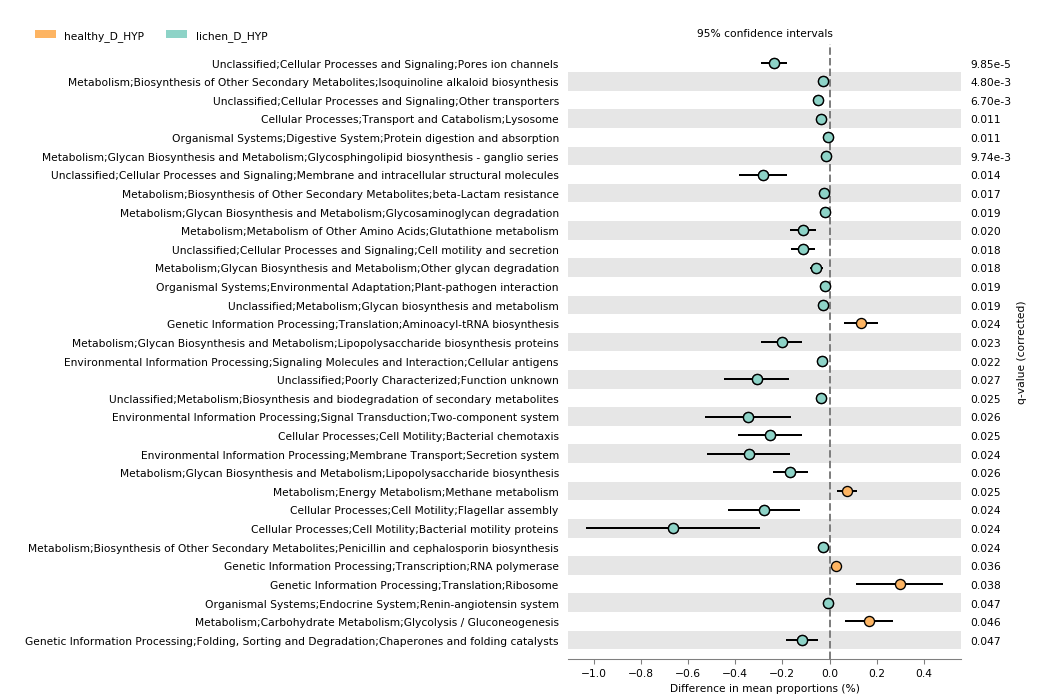

Supplement: S3 Fig — Pathway normalized abundances from PICRUSt have been analyzed by Welch’s test than corrected by multiple test (Benjamini-Hochberg). Only statistically significant pathways (q value < 0.05) have been reported. Higher mean proportions for healthy and LPP subjects have been plotted as orange or blus dots, respectively. Biochemical pathways which differ significantly in relative abundance between the dermis samples of healthy and LPP samples. The statistical analysis was performed and visualized using the STAMP package. Mean abundance (mean proportion) and difference in mean proportion for pathways showing significant difference in abundance are shown. The 95% confidence intervals and statistical significance (corrected q value) are indicated as well. (DOCX) [file pone.0269933.s005.docx]
